# VSL#3^®^ supplementation improves fatigue in long COVID: results from the DELong#3 randomized placebo-controlled trial

**DOI:** 10.3389/bjbs.2026.16993

**Published:** 2026-07-27

**Authors:** Chiara Amoroso, Beatrice Marinoni, Paola Maragno, Alessandro Rimondi, Federico Bottaro, Clorinda Ciafardini, Martina Muià, Ivanna Honcharyuk, Daniele Noviello, Bruna Caridi, Eduardo Maria Sommella, Alessandra Bandera, Andrea Gori, Marco Mantero, Francesco Blasi, Roberta Ferrucci, Alberto Priori, Francesco Strati, Maurizio Vecchi, Federica Facciotti, Flavio Caprioli

**Affiliations:** 1 Gastroenterology and Endoscopy Unit, Fondazione IRCCS Ca’ Granda Ospedale Maggiore Policlinico, Milan, Italy; 2 Department of Pathophysiology and Transplantation, Università degli Studi di Milano, Milan, Italy; 3 Department of Pharmacy, University of Salerno, Fisciano, Italy; 4 Infectious Disease Unit, Fondazione IRCCS Ca’ Granda, Ospedale Maggiore Policlinico, Milan, Italy; 5 Internal Medicine Department, Respiratory Unit and Cystic Fibrosis Center, Fondazione IRCCS Ca’ Granda Ospedale Maggiore Policlinico, Milan, Italy; 6 Department of Oncology and Hemato-oncology, Università degli Studi di Milano, Milan, Italy; 7 Neurofisiopathology Unit, Fondazione IRCCS Ca’ Granda, Ospedale Maggiore Policlinico, Milan, Italy; 8 ASST Santi Paolo e Carlo, San Paolo University Hospital, Milan, Italy; 9 Department of Scienze della salute, Università degli Studi di Milano, Milan, Italy; 10 Department of Biotechnology and Biosciences, University of Milano-Bicocca, Milan, Italy

**Keywords:** fatigue, gut microbiota, immune dysregulation, long COVID, microbiota-gut-brain axis

## Abstract

**Background:**

Long COVID is frequently characterized by persistent fatigue and impaired quality of life. Increasing evidence suggests that gut microbiota dysbiosis and immune dysregulation may contribute to symptom persistence. We evaluated the effects of the probiotic formulation VSL#3® in patients with long COVID.

**Methods:**

In this randomized, double-blind, placebo-controlled trial (ClinicalTrials.gov identifier: NCT05874089), patients with long COVID and clinically relevant fatigue received VSL#3® or placebo for 4 weeks. The primary endpoint was fatigue improvement assessed by the Chalder Fatigue Scale (CFS). Secondary and exploratory analyses included patient-reported outcomes, gut microbiota profiling, immune phenotyping, cytokine analyses, and targeted metabolomics.

**Results:**

Forty-eight patients completed the study (placebo n = 25; VSL#3® n = 23). VSL#3® supplementation significantly improved fatigue compared with placebo, with a greater reduction in CFS score (24.24% vs. 6.06%; p = 0.037) and a higher proportion of responders (68% vs. 35.7%; p = 0.019). Clinical benefit persisted after treatment discontinuation and was accompanied by improvements in selected quality-of-life and gastrointestinal symptom domains. VSL#3® supplementation was associated with selective enrichment of health-associated bacterial taxa, including *Bifidobacterium*, *Lactobacillus*, *Ruminococcus*, and *Coprococcus*, together with modulation of inflammatory and immune-related pathways. Exploratory multi-omic analyses identified coordinated associations among microbiota-derived metabolites, immune markers, and clinical outcomes.

**Conclusion:**

VSL#3® supplementation improved fatigue and selected clinical outcomes in patients with long COVID and was associated with coordinated microbiota and immune changes. These findings support further investigation of microbiota-targeted interventions in long COVID.

## Introduction

Long COVID has emerged as one of the major long-term consequences of the COVID-19 pandemic, affecting millions of individuals worldwide and posing a substantial clinical and societal burden years after acute SARS-CoV-2 infection [1–3]. Although highly heterogeneous in presentation, fatigue consistently represents one of the most prevalent and disabling manifestations, profoundly impairing quality of life, physical functioning, and return to normal daily activities [4]. Importantly, persistent symptoms frequently occur independently of acute disease severity and may remain detectable long after viral clearance [5]. Despite the growing burden of long COVID, therapeutic options remain limited, and no approved disease-modifying interventions are currently available [1].

Over the past few years, the understanding of long COVID has progressively evolved from a poorly defined post-viral condition toward a biologically complex syndrome involving persistent immune activation, metabolic dysfunction, and multisystem alterations [6–8]. Recent multiomic analyses have further reinforced this concept, identifying persistent inflammatory, metabolic, and immune signatures associated with long COVID recovery trajectories independently of acute disease severity [9]. In parallel, increasing evidence has implicated the gut microbiota as a potential contributor to long COVID pathophysiology [10]. Several studies have reported persistent alterations in gut microbial composition and function following SARS-CoV-2 infection, including depletion of health-associated commensals, reduced representation of short-chain fatty acid (SCFA)-producing bacteria, and enrichment of inflammatory microbial signatures associated with symptom persistence [10–14]. These alterations have additionally been linked to systemic inflammation, fatigue severity, sleep disturbances, neurocognitive symptoms, and impaired quality of life, supporting the hypothesis that coordinated microbiota–immune–metabolic interactions may contribute to long COVID manifestations beyond the gastrointestinal tract [9, 15–18].

This emerging framework is particularly relevant in light of the growing overlap between long COVID and other post-infectious chronic conditions, especially myalgic encephalomyelitis/chronic fatigue syndrome (ME/CFS), which share persistent fatigue, post-exertional malaise, neurocognitive dysfunction, immune dysregulation, and altered energy metabolism as common biological and clinical features [19–21]. Within this context, the microbiota-gut-brain axis has gained increasing attention as a potential mechanistic interface linking microbial dysbiosis, immune signaling, metabolism, and neurological symptoms [21].

Microbiota-targeted therapeutic approaches, including probiotics, symbiotics, and fecal microbiota transplantation, are therefore emerging as promising supportive strategies for long COVID management [15, 22]. Consistently, recent interventional studies targeting inflammatory and gut-related pathways, including vitamin K2/D3 supplementation, have shown improvement in long COVID symptom burden together with modulation of inflammatory and gut permeability markers (REF K2D3 trial) [23]. Among currently available probiotic formulations, VSL#3® was selected because of its high bacterial concentration and its well-characterized multi-strain composition, including several *Lactobacillus*, *Bifidobacterium*, and *Streptococcus* species with documented effects on intestinal barrier function, immune regulation, and microbiota homeostasis. Notably, several of these bacterial genera have been reported to be depleted in patients with COVID-19 and long COVID and have been associated with persistent inflammation and symptom burden. Furthermore, VSL#3® has shown beneficial effects in gastrointestinal and inflammatory disorders characterized by altered microbiota-host interactions, providing a biological rationale for its evaluation in long COVID [24].

Based on the growing evidence linking gut microbiota dysregulation to long COVID pathophysiology, we conducted a randomized, double-blind, placebo-controlled trial to investigate whether VSL#3® supplementation could improve fatigue and other long COVID-associated symptoms. In parallel, we performed an integrated analysis of gut microbiota composition, immune-related parameters, and microbiota-associated metabolites to explore biological pathways potentially associated with clinical response.

## Materials and methods

### Study design

This study is a single-center, randomized, double-blind, placebo-controlled trial (NCT05874089) designed to evaluate the efficacy of VSL#3®, a consortium of mixed probiotics at high concentration, in reducing fatigue and improving different features of patients’ wellbeing, microbiota composition and function, and immunological status.

Patients were consecutively recruited from the Long COVID Outpatient Clinic at IRCCS Fondazione Ca’ Granda Ospedale Maggiore Policlinico, Milan, Italy, between November 2022 and November 2023. Individuals were required to have a previous SARS-CoV-2 infection documented by molecular or antigenic nasopharyngeal swab. Demographic information, including age and sex, and history of COVID-19, including severity according to WHO classification, were obtained from hospital records. Signs and symptoms of long COVID persisting for at least 3 months after SARS-CoV-2 infection were evaluated for study inclusion. Participants were randomly assigned (1:1) to receive either VSL#3® or placebo for 4 weeks using REDCap software with block randomization stratified by sex. Double blinding was achieved by packaging supplementation and placebo in identical sealed and consecutively numbered sachets similar in appearance, smell, and taste.

The study was conducted according to the Declaration of Helsinki and Good Clinical Practice guidelines after approval by the Ethics Committee of IRCCS Fondazione Ca’ Granda Ospedale Maggiore Policlinico di Milano (approval number S62043). Written informed consent was obtained from all participants before enrolment.

### Inclusion criteria

Adult patients aged between 18 and 65 years with previous SARS-CoV-2 infection documented by molecular or antigenic nasopharyngeal swab, Chalder Fatigue Scale (CFS) score ≥4/11 in the dichotomous scoring system, and persistent long COVID symptoms for at least 3 months after infection were eligible for inclusion. Long COVID manifestations included fatigue, sleep disturbances, cognitive deficits (brain fog, impaired concentration and memory, anxiety, depression), strength deficits, arthralgias and myalgias, and gastrointestinal symptoms including reduced appetite, nausea, changes in bowel habits, and abdominal pain.

### Exclusion criteria

Exclusion criteria included use of antibiotics, probiotics, immunosuppressive/immunomodulatory drugs, opioids, or antidepressants within 30 days before enrolment; cardiovascular and pulmonary diseases with moderately severe organ dysfunction; decompensated endocrine or metabolic diseases; documented diagnosis of fibromyalgia, myalgic encephalomyelitis/chronic fatigue syndrome (ME/CFS), irritable bowel syndrome, neurological disorders, psychiatric diseases, or chronic musculoskeletal pathologies prior to SARS-CoV-2 infection; pregnancy or breastfeeding; alcohol or drug abuse; and participation in other clinical trials within 30 days before enrolment. Use of probiotics, antibiotics, immunosuppressive/immunomodulatory drugs, opioids, or antidepressants during the study was considered prohibited medication. Major dietary modifications during the trial were considered protocol violations.

### Procedures

VSL#3® (Actial Farmaceutica) is a consortium of eight live freeze-dried lactic acid bacteria and bifidobacteria (*Streptococcus thermophilus* BT01; *Bifidobacterium breve* BB02; *Bifidobacterium animalis subsp. lactis* BL03; *Bifidobacterium animalis subsp. lactis* BI04; *Lactobacillus acidophilus* BA05; *Lactobacillus plantarum* BP06; *Lactobacillus paracasei* BP07; and *Lactobacillus helveticus* BD08) at high concentration (450 billion CFU/sachet). Placebo sachets were identical in appearance and composition except for the absence of probiotics.

After screening evaluation, study procedures were performed at baseline (t0), after 4 weeks of supplementation (t4), and after an additional 4-week follow-up period (t8). Participants received two sachets/day for 28 days. Compliance was assessed by sachet counting and considered adequate when ≥80% of sachets were consumed. Fecal samples were collected for microbiome profiling and metabolomic analyses, including short-chain fatty acids (SCFAs) and neuromodulators. Blood samples were collected for immune phenotyping by multiparametric flow cytometry and serum cytokine profiling by multiplex Luminex assay.

### Study endpoints

The primary endpoint was reduction in fatigue after 4 weeks of supplementation as measured by the Chalder Fatigue Scale (CFS). Secondary outcomes included variations in Hospital Anxiety and Depression Scale (HADS), Short Form-36 Health Survey (SF-36), Structured Assessment of Gastrointestinal Symptoms (SAGIS), Symptoms Check List-12 (SCL-12), Karnofsky Performance Status (KPS), and Visual Analogue Scale (VAS) scores. Additional exploratory endpoints included modulation of gut microbiota composition, circulating immune parameters, short-chain fatty acids (SCFAs), and neuromodulators.

### Structured questionnaires

Structured questionnaires were completed at baseline (t0), after supplementation (t4), and at follow-up (t8), and included CFS, HADS, SF-36, SCL-12, KPS, VAS, and SAGIS questionnaires. Detailed description of questionnaires and scoring systems is reported in the Supplementary Methods.

### Sample size calculation

Assuming a placebo effect of 30% on fatigue reduction and a probiotic efficacy of 60% on the primary endpoint, 23 patients per arm were estimated to provide 80% statistical power with a two-sided significance level of 0.05. Considering an estimated dropout rate of 15%, at least 53 patients were planned for recruitment.

### Multiplex serum biomarker analysis

Multiplex analysis of circulating cytokines and chemokines was performed using a 45-plex Human XL Cytokine Discovery Luminex panel (R&D Systems) according to the manufacturer’s instructions. Samples were acquired on a Luminex 200SD instrument and analyzed using xPONENT software v4.2. Luminex analyses were performed on a selected subset of patients (n = 28; placebo n = 15; VSL#3® n = 13). The complete list of analytes included in the panel is summarized in Supplementary Table S1.

### Cytofluorimetric analyses

Peripheral blood mononuclear cells (PBMCs) collected at baseline and t4 were isolated by Ficoll density-gradient centrifugation. Cells were stained using fluorochrome-conjugated monoclonal antibodies listed in Supplementary Table S2. For intracellular cytokine staining, cells were stimulated for 3 h at 37 °C with PMA (50 ng/mL), ionomycin (1 μg/mL), and Brefeldin A (10 μg/mL), then fixed and permeabilized using Cytofix/Cytoperm (BD Biosciences). Samples were acquired on a FACSLyrics flow cytometer (BD Biosciences) and analyzed using FlowJo software v10.8 after exclusion of doublets and non-viable cells. Flow cytometric analyses were performed on the entire study cohort.

### 16S rRNA gene sequencing and microbiota analysis

Fecal samples collected at t0 and t4 were stored at −80 °C until DNA extraction. DNA extraction, 16S rRNA gene amplification, purification, library preparation and pair-end sequencing on the Illumina MiSeq platform were performed as previously described (V3-V4 regions, 300 bp paired end) [25]. Reads were pre-processed using the MICCA pipeline (v.1.7.2) (https://micca.readthedocs.io/en/latest/#) [26]. Forward and reverse primers trimming and quality filtering were performed using micca trim and micca filter, respectively. Filtered sequences were denoised using the UNOISE [27] algorithm implemented in micca otu to determine true biological sequences at the single nucleotide resolution by generating amplicon sequence variants (ASV). Bacterial ASVs were taxonomically classified using micca classify and the Ribosomal Database Project (RDP) Classifier v2.13 [28]. Multiple sequence alignment (MSA) of 16S rRNA gene sequences was performed using the Nearest Alignment Space Termination (NAST) algorithm implemented in micca msa [29]. Phylogenetic trees were inferred using micca tree [29, 30]. Sampling heterogeneity was reduced rarefying samples at the depth of the less abundant sample using micca tablerare. Alpha (within-sample richness) and beta-diversity (between-sample dissimilarity) estimates were computed using the phyloseq R package [31]. Permutational multivariate analysis of variance (PERMANOVA) test was performed using the adonis2 function in the vegan R package with 999 permutations. ASVs differential abundance testing was carried out using the R package DESeq2 using the non-rarefied data [31, 32].

### Metabolomic analyses

Fecal short-chain fatty acids (SCFAs), fecal neuromodulators, and serum neuromodulators were quantified using targeted chromatographic approaches. Metabolomic analyses were performed on a subset of patients, selected on the basis of the material availability to perform the analyses (n = 30; placebo n = 16; VSL#3® n = 14).

Targeted quantification of dopamine and serotonin in serum and fecal samples was performed by ultra-high-performance liquid chromatography coupled with tandem mass spectrometry (UHPLC-MS/MS). Briefly, serum proteins were precipitated using ice-cold acetonitrile, whereas fecal samples were extracted using 50% methanol solution in the presence of deuterated internal standards (dopamine-d4 and serotonin-d4). UHPLC-MS/MS analyses were carried out on a Shimadzu Nexera UHPLC system coupled to a triple quadrupole LCMS-8050 mass spectrometer equipped with an electrospray ionization source. Chromatographic separation was performed on an HSS T3® C18 column (150 × 2.1 mm, 1.8 μm; Waters®) using water and acetonitrile containing 0.1% formic acid as mobile phases. Analyses were conducted in scheduled multiple reaction monitoring (sMRM) mode.

SCFA quantification was performed by gas chromatography-mass spectrometry (GC-MS) according to a previously validated protocol with minor modifications based on Zhu et al [33]. Briefly, freeze-dried fecal samples were acidified, extracted with methyl tert-butyl ether (MTBE), and supplemented with deuterated butyric acid as internal standard. GC-MS analyses were performed using an Agilent 8890 GC system coupled with a 5977B mass selective detector operating in selected ion monitoring (SIM) mode. Chromatographic separation was achieved using a J&W DB-WAX GC column (30 m × 0.25 mm × 0.25 μm; Agilent Technologies).

Data acquisition and processing were performed using MassHunter Qualitative Analysis software (Agilent Technologies). Correlation analyses integrating metabolomic, microbiota, immune, and clinical datasets were performed using Spearman’s correlation tests in R environment (v4.4.1).

### Statistical analysis

All statistical analyses were performed using R v4.4.1 and GraphPad Prism v10.2.3. Parametric and non-parametric tests were applied according to data distribution, including ANOVA, t-test, Wilcoxon signed-rank test, Mann–Whitney U test, and Spearman’s correlation analyses. Primary endpoint analyses were performed both in the intention-to-treat and per-protocol populations.

### Data availability

16S rRNA gene sequencing data are available in the European Nucleotide Archive (https://www.ebi.ac.uk/ena) under accession number PRJEB78610.

## Results

### Study population and baseline characteristics

**FIGURE 1:**
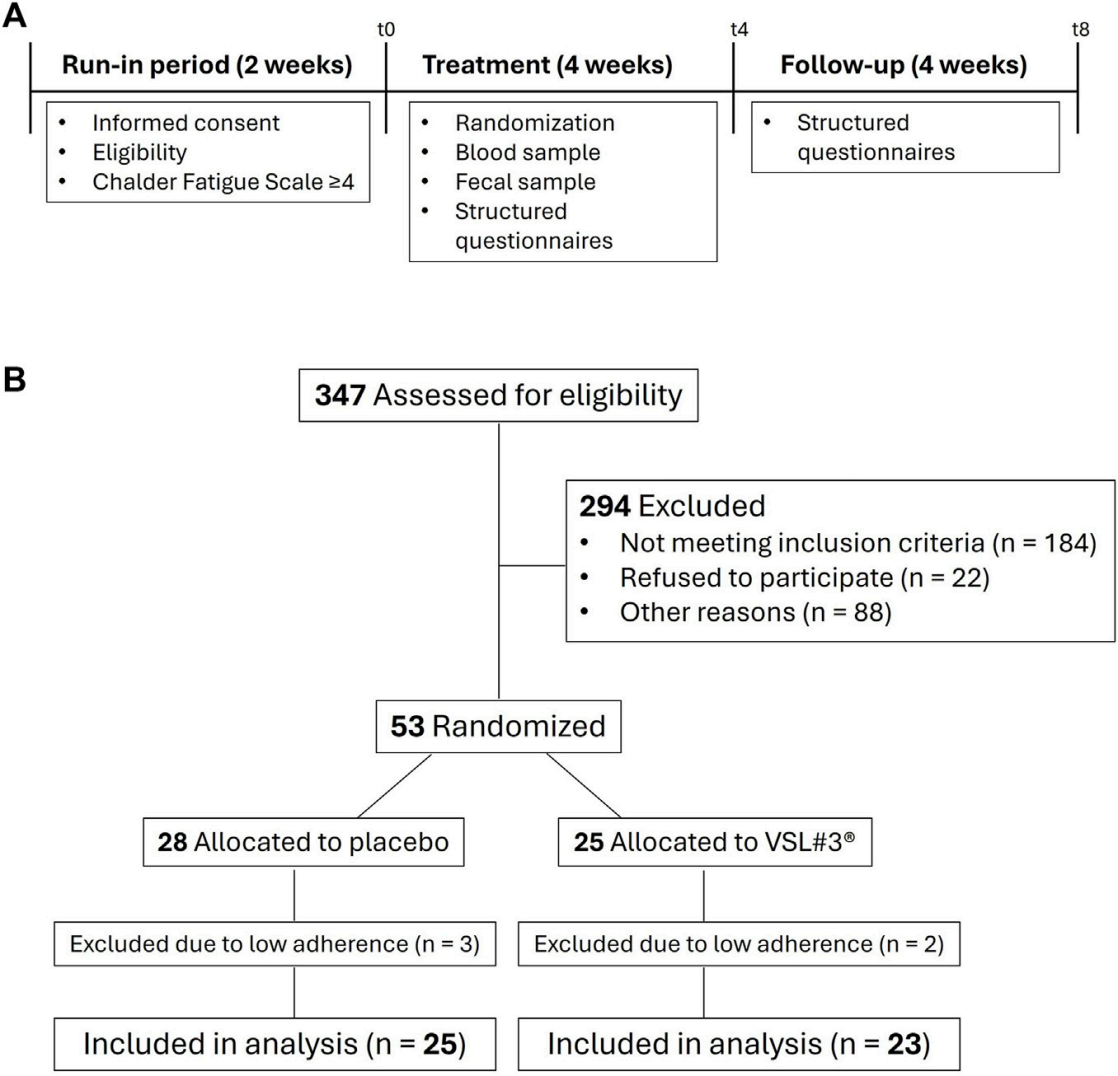
Study design and participant flow. **(A)** Schematic representation of the study design, including run-in, treatment, and follow-up periods, with timing of sample collection and clinical assessments. **(B)** Flow diagram of participant enrolment, randomization, and analysis.

Between November 2022 and November 2023, a total of 347 individuals with a history of SARS-CoV-2 infection and persistent symptoms were screened for eligibility at the Long COVID outpatient clinic of Policlinico Hospital (Milan, Italy). Among these, 53 patients met the inclusion criteria and were enrolled in the study (Figures 1A,B). Overall, 294 patients were excluded after screening. The main reasons for exclusion were relevant comorbidities (47.5%), absence of clinically relevant fatigue (35.8%), ongoing antidepressant treatment (7.9%), refusal to participate (8.3%), and concomitant probiotic use (0.4%). Among comorbidities, the most frequent causes of exclusion were cardiovascular diseases, psychiatric disorders, oncological diseases, musculoskeletal disorders, neurological disorders, pulmonary diseases, metabolic diseases, immunological diseases, immunosuppression, and the presence of multiple concomitant conditions. All enrolled participants reported persistent symptoms for at least 3 months after acute infection, consistent with current definitions of post-acute sequelae of SARS-CoV-2 infection. Following randomization, 28 patients were allocated to the placebo group and 25 to the VSL#3® group. Five participants were excluded due to low adherence to the study protocol, resulting in a final study population of 48 individuals (25 placebo, 23 VSL#3®) (Figures 1A,B). Baseline demographic and clinical characteristics of the study population are reported in Table 1. The cohort included 24 females and 24 males, with a median age of 54 years and a median body mass index (BMI) of 25. No significant differences were observed between the placebo and VSL#3® groups in terms of age, sex distribution, BMI, or clinical history (Table 1).

**TABLE 1:** Baseline clinical characteristics of the enrolled subjects.

Clinical parameter	Total (n = 48)	Placebo (n = 25)	VSL#3® (n = 23)	p-value
Male/Female, n (%)	24/24 (50)	13/12 (52)	11/12 (47.8)	>0.9
Age at enrolment, years, median (IQR)	54 (48; 60)	55 (48; 62)	53 (47; 59)	0.3524
Body mass index BMI, median (IQR)	25 (22; 29)	25 (24; 29)	24 (22; 27)	0.2763
Current smoker, n (%)	8 (16.7)	4 (16)	4 (17.4)	>0.99
Current drinker, n (%)	17 (35.4)	12 (48)	5 (21.7)	0.11
Hospitalization, n (%)	24 (50)	13 (52)	11 (47.8)	>0.99
Hospitalization duration, days median (IQR)	1.5 (0; 19.5)	0 (0; 21)	7 (0; 18.5)	0.6117
ICU, n (%)	7 (14.6)	4 (16)	3 (13)	>0.99
IOT, n (%)	7 (14.6)	4 (16)	3 (13)	>0.99
Intestinal pneumonia, n (%)	26 (54.2)	13 (12)	13 (10)	0.9807
Abx_acute infection, n(%)	25 (52.1)	12 (48)	13 (56.5)	0.7632
Antivirals_acute infection, n (%)	13 (27.1)	4 (16)	9 (39.1)	0.1398
Acute_GI symptoms, n (%)	19 (39.5)	10 (40)	9 (39.1)	>0.99
Duration of LC symptoms, months median (IQR)	30 (21.25; 34)	28 (17; 34)	34 (27; 35)	0.07432
LC symptoms Brain fogging, n (%) Gastrointestinal, n (%) Concentration, n (%) Memory loss, n (%) Dyspnea, n (%) Chest pain, n (%) Cough, n (%) Anosmia, n (%) Dysgeusia, n (%) Headache, n (%) Rhinitis, n (%) Alopecia, n (%) Poor appetite, n (%) Dizziness, n (%) Myalgias, n (%) Joint pain, n (%)	46 (95.8) 25 (52.1) 44 (91.7) 35 (72.9) 24 (50) 6 (12.5) 4 (8.3) 2 (4.2) 0 11 (22.9) 2 (4.2) 11 (22.9) 9 (18.8) 18 (37.5) 33 (68.8) 33 (68.8)	24 (95.6) 14 (56) 22 (88) 17 (68) 11 (44) 2 (8) 2 (8) 2 (8) 0 7 (28) 1 (4) 5 (20) 3 (12) 10 (40) 17 (68) 16 (69.6)	22 (95.6) 11 (47.8) 22 (95.7) 18 (78.3) 13 (56.5) 4 (17.4) 2 (4.3) 0 0 4 (17.4) 1 (4.3) 6 (26.1) 6 (26.1) 8 (34.8) 16 (69.6) 17 (73.9)	>0.99 0.7817 0.6631 0.6354 0.5634 0.5851 >0.99 0.5075 1 0.5962 >0.99 >0.99 0.4616 0.9405 >0.99 0.6683
Sweating, n (%)	6 (13)	3 (12)	3 (13)	>0.99

Overall, 50% of participants had a history of hospitalization during the acute phase of COVID-19, and a substantial proportion reported gastrointestinal symptoms both during the acute infection and at the time of Long COVID evaluation (Table 1). The median duration of persistent symptoms was 30 months, with comparable distribution between the two study groups (Table 1). The spectrum of Long COVID symptoms among the participants included cognitive impairment (“brain fog”) (95.8%), reduced concentration (91.7%), memory loss (72.9%), musculoskeletal pain (68.8%), gastrointestinal symptoms (52.1%), dyspnea (50%), dizziness (37.5%), hair loss (22.9%), poor appetite (18.8%), chest discomfort (12.5%), chronic cough (8.3%), and rhinitis (4.2%) (Table 1). All participants reported the presence of more than four symptoms in addition to fatigue. Analyses of clinical outcomes were performed on the full study population. For microbiota, immune, and metabolomic analyses, subsets of patients were included based on sample availability; the number of samples analyzed for each assay is indicated in the corresponding figures. No clinically relevant adverse events were reported during the study period.

### VSL#3® supplementation improves fatigue and patient-reported outcomes in long COVID patients

**FIGURE 2:**
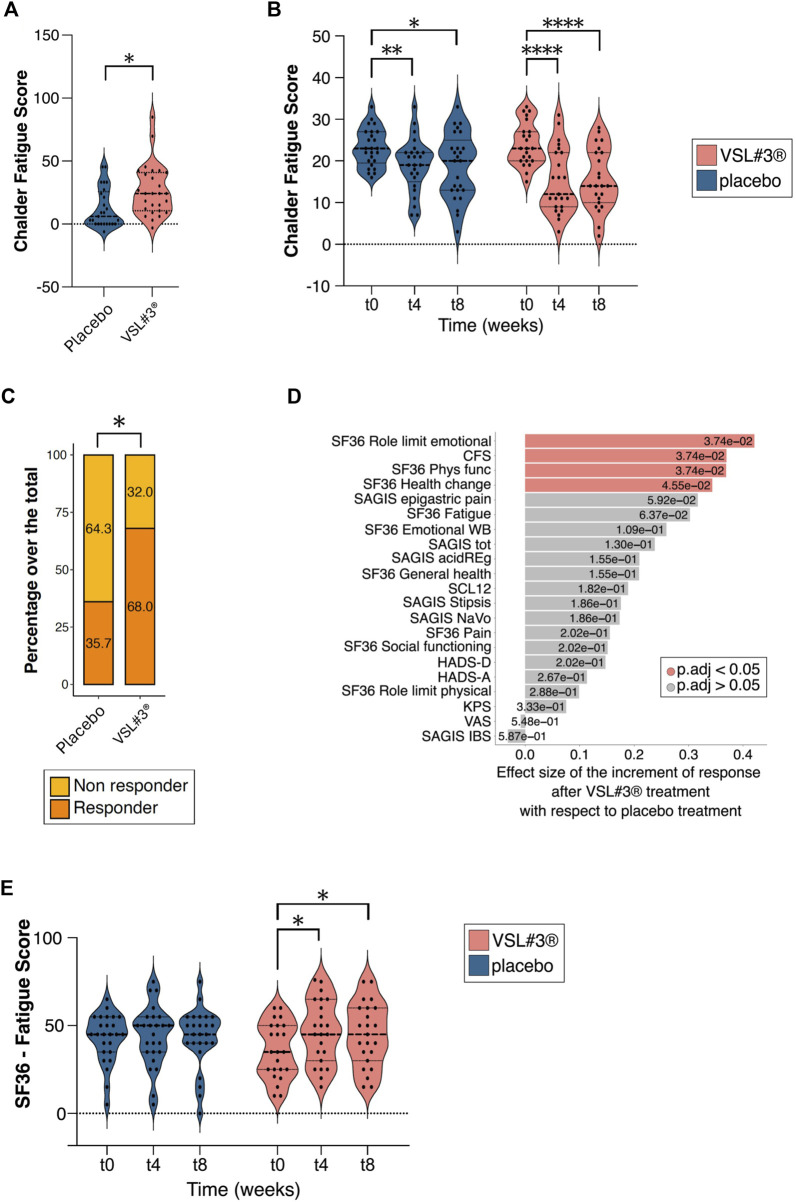
Effects of VSL#3® supplementation on fatigue and patient-reported outcomes in long COVID patients. **(A)** Variation in Chalder Fatigue Scale (CFS) score in placebo and VSL#3®-treated patients. **(B)** CFS scores over time in placebo (blue) and VSL#3®-treated (red) patients. **(C)** Percentage of responders in placebo and VSL#3®-treated patients. **(D)** Effect size of changes in patient-reported outcomes in VSL#3®-treated patients compared to placebo; parameters with adjusted p-values <0.05 are highlighted. Statistical analysis has been performed by Mann Whitney test, one tailed. **(E)** SF-36 fatigue scores at baseline (t0), end of treatment (t4), and follow-up (t8) in placebo (blue) and VSL#3®-treated (red) patients. Data are shown as individual values with distribution plots. Statistical analysis has been performed by two-ways ANOVA. *p < 0.05; **p < 0.01; ****p < 0.0001.

The primary endpoint of the study was the change in fatigue, assessed by the Chalder Fatigue Scale (CFS), after 4 weeks of supplementation. A significant reduction in fatigue was observed in patients receiving VSL#3® compared to placebo from baseline (t0) to the end of treatment (t4) (Figures 2A,B; Table 2). In line with this, the change in CFS score was greater in the VSL#3® group compared to placebo (24.24 vs. 6.06, p = 0.037) (Figure 2A). This improvement was maintained at follow-up (t8) in the VSL#3® group, whereas no significant changes were observed in the placebo group (Figure 2B). In parallel, the proportion of responders was higher among VSL#3®-treated patients compared to placebo (68% vs. 35.7%, p = 0.019) (Figure 2C).

**TABLE 2:** Statistical analysis of patient-reported outcomes.

Variable	Placebo (n = 25)	VSL#3® (n = 23)	p-value^§^
CFS Likert variation %	6.06 (0; 24.24)	24.24 (10.61; 40.91)	0.03738
(SF)-36 Fatigue variation %	0 (−5; 5)	10 (0; 15)	0.0637
Physical functioning variation %	0 (−10; 5)	5 (0; 17.5)	0.03738
Role limitation due to emotional problems variation %	0 (0; 0)	32 (0; 67)	0.03738
Role limitation due to physical health variation %	0 (0; 25)	0 (0; 25)	0.288167
Social functioning variation %	0 (0; 13)	12 (0; 25)	0.202125
Emotional wellbeing variation %	0 (−4; 4)	4 (0; 16)	0.1092
Health change variation %	0 (−25; 0)	0 (0; 50)	0.045518
General health variation %	0 (−5; 0)	5 (−5; 15)	0.1554
Pain variation %	0 (−10; 12)	0 (0; 17)	0.202125
SAGIS	​	​	​
Total variation %	0 (−2.27; 3.41)	3.41 (−0.57; 7.39)	0.130463
Epigastric pain variation %	0 (−3.57; 3.57)	7.14 (0; 10.71)	0.05922
IBS Variation %	0 (−4.17; 8.33)	4.17 (−2.08; 4.17)	0.587
Acid regurgitation variation %	0 (0; 8.33)	8.33 (0; 16.67)	0.1554
Nausea and vomit variation %	0 (−6.25; 0)	0 (−3.13; 6.25)	0.185769
Constipation variation %	0 (0; 0)	0 (0; 12.5)	0.185769
(SCL)-90 Somatization variation %	4.17 (−2.08; 8.33)	8.33 (1.04; 10.42)	0.182318
HADS -A variation %	0 (0; 9.52)	4.76 (0; 9.52)	0.266824
-D variation %	0 (0; 9.52)	4.76 (0; 14.29)	0.202125
KPS	0 (0; 0)	0 (0; 10)	0.332684
VAS	0 (−20; 0)	−10 (−20; 0)	0.5481

For each questionnaire, data are expressed as the median (first and third quartile) of the change in score between T0 and T4 in placebo and VSL#3®-treated groups. P-values indicate differences between groups. Values are reported as median (first quartile, third quartile). § P-values were calculated using the Mann-Whitney test.

Patient-reported outcomes showed patterns consistent with the primary endpoint. Improvements in quality of life, assessed by SF-36, were observed in the VSL#3® group at week 4; however, these changes did not reach statistical significance (Figure 2D; Table 2). Longitudinal analysis of SF-36 fatigue scores showed an improvement from baseline to week 4 that was maintained at follow-up (t8) in the VSL#3® group, whereas no consistent changes were observed in the placebo group (Figure 2E). Significant improvements were observed in specific symptom domains, including epigastric pain and gastrointestinal disturbances, as well as in measures of emotional wellbeing (adjusted p-values <0.05; Supplementary Figures S1–S3). No statistically significant differences were detected across other clinical questionnaires, although numerical improvements were observed in the VSL#3® group across several measures.

Overall, these findings demonstrate that VSL#3® supplementation significantly improved fatigue in long COVID patients, with benefits persisting up to 4 weeks after treatment discontinuation, accompanied by improvements in selected patient-reported outcomes.

### VSL#3® supplementation is associated with compositional changes in the gut microbiota of long COVID patients

**FIGURE 3:**
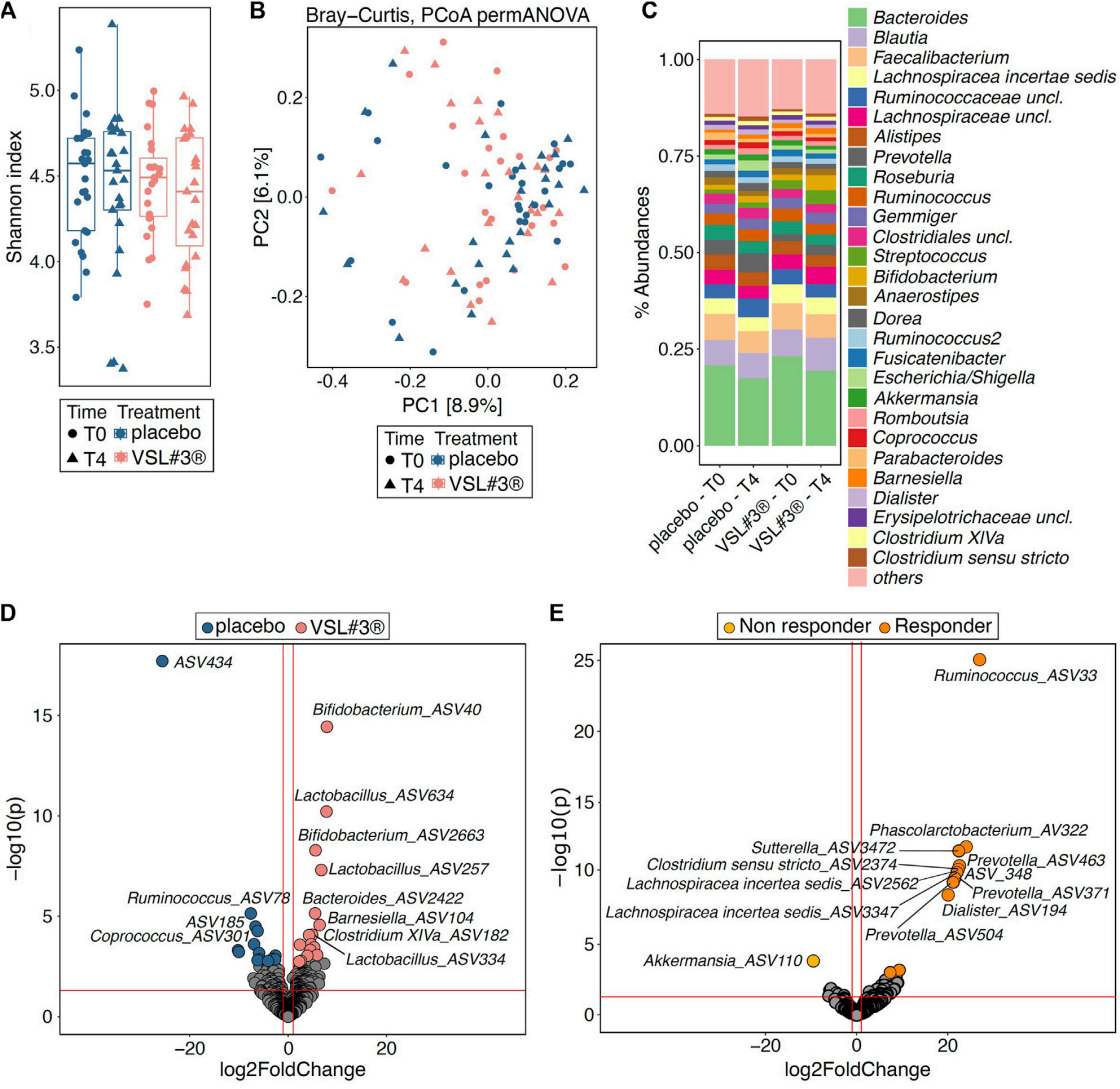
VSL#3® supplementation is associated with compositional changes in the gut microbiota. **(A,B)** Alpha (**A**, Shannon index) and beta (**B**, Bray-Curtis dissimilarity) diversity in placebo (blue) and VSL#3®-treated (red) patients at baseline (t0) and at the end of treatment (t4). **(C)** Relative abundance of bacterial taxa in placebo and VSL#3®-treated patients. **(D,E)** Differentially abundant taxa identified at t4 between placebo and VSL#3®-treated patients **(D)** and between clinical responders and non-responders within the VSL#3® group **(E)**. Statistical analysis has been performed by DESeq2. Only the ASVs with a FDR < 0.05 and an absolute log2FC > 1 are colored, and the name is reported only for the subset with a FDR < 0.01.

Gut microbiota alterations have been reported in patients with COVID-19 and may persist after resolution of the acute infection [10, 11]. In the present study, no significant differences in microbial diversity were observed between placebo and VSL#3®-treated patients at baseline (t0) or at the end of treatment (t4), as assessed by alpha diversity (Shannon index) and beta diversity (Bray-Curtis dissimilarity) (Figures 3A,B).

Despite the absence of global changes in community structure, differential abundance analysis identified specific taxa enriched in VSL#3®-treated patients at t4, including members of the *Bifidobacterium*, *Lactobacillus*, *Streptococcus*, and *Barnesiella* genera (Figures 3C,D). Several of these taxa correspond to strains contained in the VSL#3® formulation (Supplementary Table S3).

Within the VSL#3® group, clinical responders exhibited distinct microbial signatures compared to non-responders, with enrichment of taxa including *Bacteroides*, *Ruminococcus*, *Coprococcus*, *Phascolarctobacterium*, *Sutterella*, *Prevotella*, *Holdemanella*, *Akkermansia*, and *Clostridium* cluster IV (Figure 3E).

Functional prediction analysis suggested differences in microbial metabolic pathways following VSL#3® supplementation, including enrichment of glycine, threonine, and methionine metabolism (KEGG pathway ko00260), a pathway utilized by commensal bacteria and potentially linked to microbial metabolic activity^36^ (Supplementary Figure S4). Conversely, no consistent enrichment of microbiome-related pathways was observed in placebo-treated individuals (Supplementary Figure S4).

Overall, these findings indicate that VSL#3® supplementation is associated with selective compositional changes in the gut microbiota, particularly among clinical responders, in the absence of major shifts in overall microbial diversity.

### VSL#3® supplementation is associated with modulation of immune parameters in long COVID patients

**FIGURE 4:**
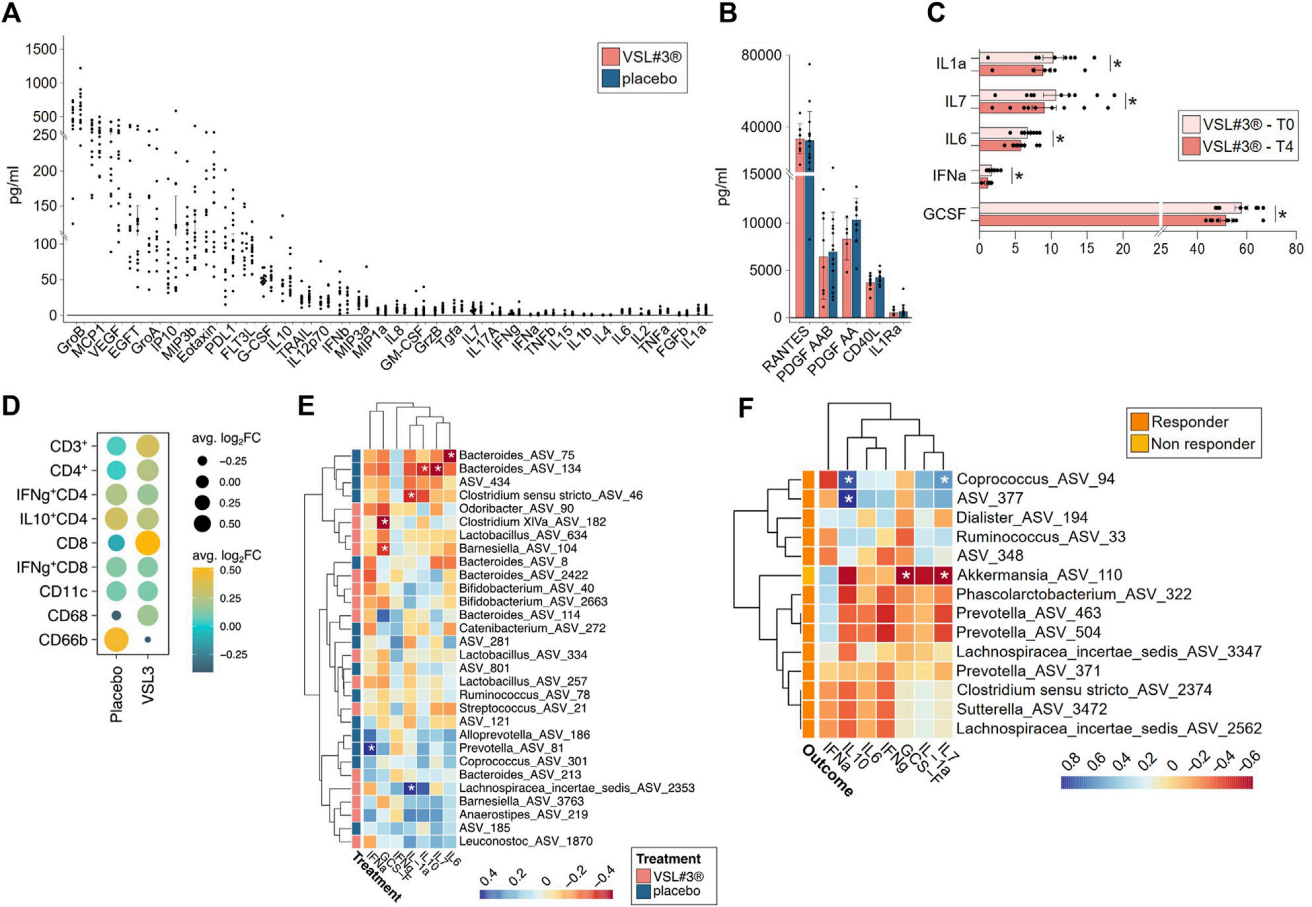
VSL#3® supplementation is associated with modulation of immune parameters and microbiota-immune interaction patterns in long COVID patients. **(A,B)** Concentration of circulating cytokines and chemokines measured by Luminex analysis in serum samples from placebo (blue symbols) and VSL#3®-treated (pink symbols) patients at t4. Cytokines and chemokines below **(A)** and above **(B)** 1500 pg/mL are shown separately. **(C)** Serum levels of cytokines associated with inflammatory and immune activation in VSL#3®-treated patients at baseline (t0, light pink symbols) and end of treatment (t4, dark pink symbols). Cytokine analyses (panels **A–C**) were performed on a selected subset of patients with available paired serum samples at t0 and t4 (n = 28; placebo n = 15, VSL#3® n = 13). **(D)** Balloon plot showing scaled integrated frequencies of the indicated immune cell populations in placebo and VSL#3®-treated patients following flow cytometry-based immune phenotyping analysis. Immune phenotyping analyses were performed on the full study cohort. **(E,F)** Heatmaps showing Spearman’s rho correlations between significantly enriched bacterial taxa and immune parameters in **(E)** placebo and VSL#3®-treated patients at t4 and **(F)** responder and non-responder patients within the VSL#3® group at t4. Significant correlations (p < 0.05) are indicated by an asterisk (*). Statistical analysis has been performed by t- test, two tailed.

The gut microbiota has been shown to influence host immune responses [34]. To investigate the impact of VSL#3® supplementation on systemic immune parameters, serum cytokine and chemokine profiles were assessed by multiplex analysis. Overall, circulating cytokine and chemokine levels were comparable between placebo and VSL#3®-treated patients (Figures 4A,B). Within the VSL#3® group, reductions in IL1α, IL6, IL7, IFNα, and G-CSF levels were observed at the end of treatment (t4) compared with baseline (Figure 4C), suggesting a partial attenuation of persistent low-grade immune activation associated with long COVID. However, no significant differences between treatment groups were detected at t4. Therefore, these findings should be considered exploratory and interpreted with caution.

In parallel, immune cell phenotyping revealed changes in cellular immune populations following VSL#3® supplementation, including increased proportions of CD8^+^ T cells and CD68^+^ macrophages together with reduced CD66b+ granulocytes at t4 compared to baseline (Figure 4D), suggesting a remodelling of innate and adaptive immune responses following supplementation.

To further explore the relationship between microbiota composition and immune responses, correlation analysis was performed. Distinct association patterns between microbial taxa and immune parameters were observed in VSL#3®-treated patients compared to placebo (Figure 4E). In particular, taxa enriched in the VSL#3® group were associated with members of the Lachnospiraceae family and with IL1α levels, a cytokine involved in cellular stress and inflammatory signaling [35], whereas in placebo-treated individuals, *Prevotella* spp. were associated with IFNα, a cytokine involved in antiviral and pro-inflammatory immune responses [36]. In addition, *Coprococcus*, a commensal bacterium [37], showed positive correlations with IL10 and IL7 in responder patients, suggesting a potential link between commensal microbial signatures and pathways involved in immune regulation and persistence of low-grade inflammation [25, 38, 39]. Conversely, in non-responders, *Akkermansia* showed negative correlations with IL17 and G-CSF (Figure 4F), suggesting distinct microbiota-immune interaction patterns associated with altered inflammatory signaling.

Collectively, these findings indicate that VSL#3® supplementation is associated with remodeling of inflammatory and immune-related pathways, accompanied by distinct microbiota-immune interaction patterns in long COVID patients.

### Microbiota-derived metabolites and neuromodulators are associated with clinical response to VSL#3® supplementation

**FIGURE 5:**
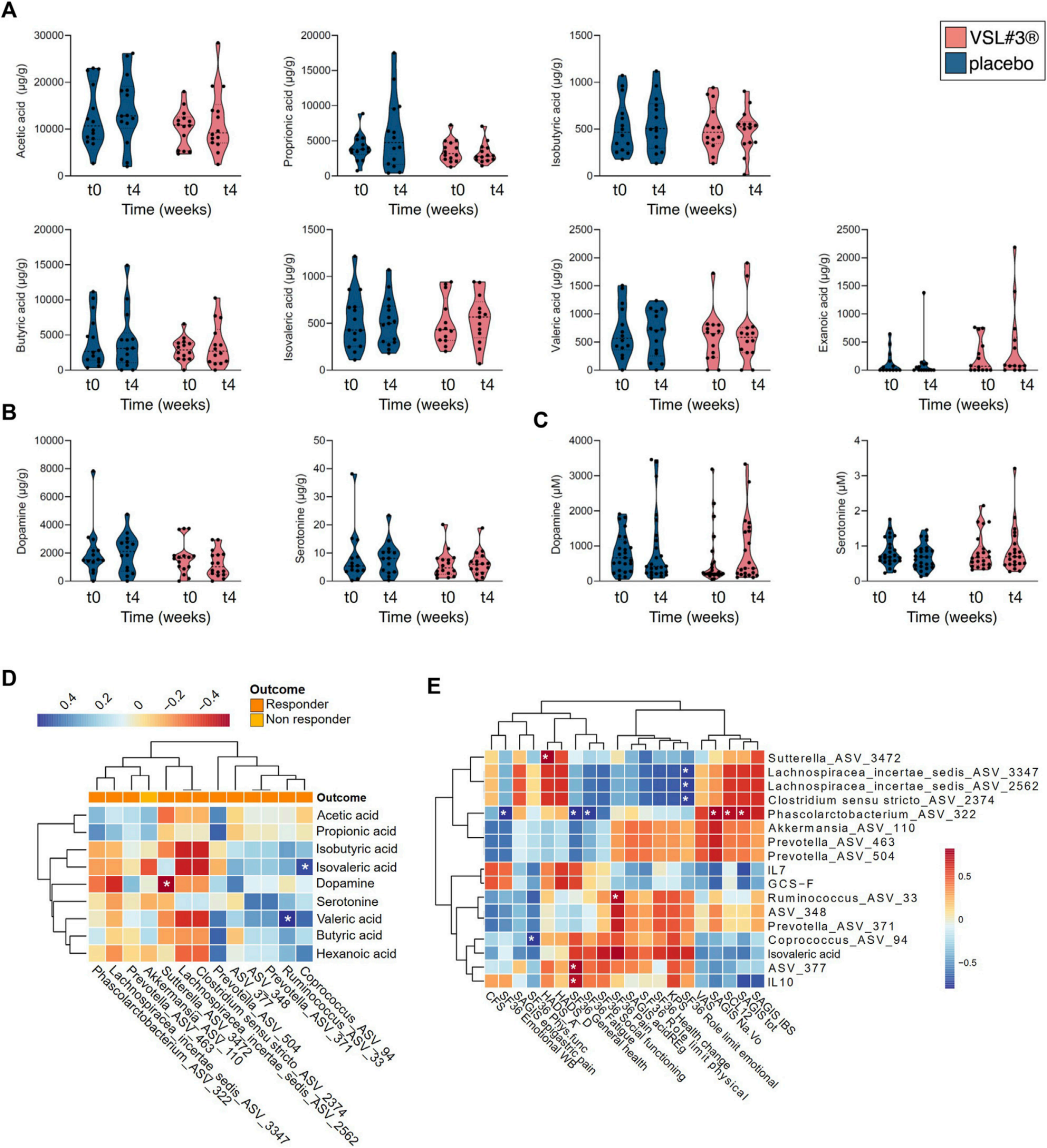
Microbiota-derived metabolites and neuromodulators are associated with clinical response to VSL#3® supplementation. **(A)** Fecal concentrations (μg/g) of SCFAs measured by targeted mass spectrometry-based analyses in placebo (blue symbols) and VSL#3®-treated (red symbols) patients. **(B)** Fecal concentrations (μg/g) of dopamine and serotonin measured by targeted mass spectrometry-based analyses in placebo (blue symbols) and VSL#3®-treated (red symbols) patients. **(C)** Serum concentrations (μM) of dopamine and serotonin in placebo (blue symbols) and VSL#3®-treated (red symbols) patients. Metabolomic analyses (panels **A–C**) were performed on a selected subset of patients (n = 30; placebo n = 16; VSL#3® n = 14). **(D)** Heatmap showing Spearman’s rho correlations between microbiota-derived metabolites and bacterial ASVs enriched in responder patients. **(E)** Correlation plot showing Spearman’s correlation coefficients computed between, on one side, metabolite levels, cytokine concentrations, and ASVs differentially enriched between responder and non-responder groups at T4 and, on the other side, questionnaire scores at T4. Correlations with a nominal p-value <0.05 are indicated by an asterisk (*). Statistical analysis has been performed by t- test, two tailed.

To further investigate the relationship between gut microbiota remodeling and clinical improvement, fecal SCFAs together with fecal and serum neuromodulators were evaluated by targeted metabolomic analyses. Overall, fecal SCFA and neuromodulator levels showed high inter-individual variability and no major differences between placebo and VSL#3®-treated patients over time (Figure 5A). Similarly, fecal dopamine and serotonin concentrations did not show consistent differences between treatment groups (Figure 5B). Serum dopamine and serotonin levels also showed high variability across patients, without significant differences between placebo and VSL#3®-treated groups over time (Figure 5C).

However, exploratory analyses in responder patients identified associations between specific microbiota-derived metabolites and bacterial taxa enriched following supplementation. In particular, correlation analyses performed in responder patients revealed positive associations between isovaleric acid and *Coprococcus*, as well as between valeric acid and *Ruminococcus* (Figure 5D), two commensal bacterial genera associated with microbial metabolic activity and gut ecosystem balance [37, 40]. Furthermore, exploratory correlation analyses integrating metabolite levels, cytokine concentrations, ASVs differentially enriched between responder and non-responder groups, and questionnaire scores at T4 revealed coordinated microbiota-immune-metabolic patterns associated with clinical outcomes (Figure 5E).

Collectively, these findings suggest that clinical improvement following VSL#3® supplementation is associated with coordinated microbiota-immune-metabolic interaction patterns potentially involved in gut-brain communication.

## Discussion

Fatigue remains one of the most prevalent and disabling manifestations of long COVID, substantially affecting daily activities and quality of life. Five years after the beginning of the COVID-19 pandemic, long COVID continues to represent a major unmet clinical challenge, with no approved disease-modifying therapies currently available and only limited evidence supporting existing interventions [5, 41, 42].

In this randomized, double-blind, placebo-controlled trial, we investigated the effects of VSL#3® supplementation in patients with long COVID, specifically targeting fatigue as the primary endpoint. In our cohort, VSL#3® supplementation significantly improved fatigue, as assessed by the Chalder Fatigue Scale, with a higher proportion of clinical responders compared to placebo-treated patients (Figures 2A–C). Notably, the clinical benefit persisted up to 4 weeks after treatment discontinuation, suggesting a sustained effect beyond the supplementation period. In parallel, improvements were also observed in physical functioning, general wellbeing, health-related quality of life, and gastrointestinal symptoms (Figures 2D,E), further supporting the potential relevance of gut-directed interventions in long COVID patients.

The biological mechanisms underlying long COVID remain incompletely understood and are likely heterogeneous across patients. Among the proposed pathogenic mechanisms, increasing evidence supports a role for gut microbiota dysbiosis in long COVID pathophysiology. Previous studies have consistently reported persistent gut microbiota alterations in both acute COVID-19 and long COVID patients, including depletion of health-associated commensals such as *Bifidobacterium* and *Lactobacillus*, enrichment of opportunistic pathogens, and reduction of butyrate-producing bacteria, often correlating with inflammatory markers and symptom persistence [11–14, 43]. In agreement with these observations, VSL#3® supplementation in our cohort was associated with enrichment of health-associated bacterial genera, including *Bifidobacterium*, *Lactobacillus*, *Barnesiella*, and *Streptococcus*, together with SCFA-associated taxa such as *Ruminococcus*, *Coprococcus*, and *Clostridium* cluster IV (Figures 3D,E). These findings occurred in the absence of major shifts in alpha and beta diversity, supporting the concept that specific alterations in microbial composition and associated metabolic functions may be more informative than global diversity metrics in explaining clinical outcomes [44]. In recent years, microbiota-targeted therapeutic approaches, including probiotics, symbiotics, and fecal microbiota transplantation, have emerged as potential supportive strategies for long COVID management [17, 45]. In this context, VSL#3® represents a probiotic formulation already widely available in Europe and the United States, potentially facilitating the clinical translation of microbiota-based interventions. It should be noted that several of the bacterial taxa enriched following VSL#3® supplementation, including *Bifidobacterium*, *Lactobacillus*, and *Streptococcus*, correspond to genera contained in the administered formulation (Supplementary Table S3). Their increased relative abundance at t4 may therefore partly reflect transient detection of the supplemented organisms rather than durable remodeling of the resident microbiota, a distinction that cannot be fully resolved by 16S rRNA gene profiling. Nevertheless, clinical responders were additionally characterized by the enrichment of commensal taxa not present in the formulation, such as *Bacteroides*, *Ruminococcus*, *Coprococcus*, and *Clostridium* cluster IV (Figure 3E), suggesting that the observed changes may extend beyond the simple persistence of administered strains. Moreover, the maintenance of clinical benefit 4 weeks after treatment discontinuation argues against a purely transient effect. However, given the relatively short follow-up period, it remains unclear whether these compositional changes are durable, and longer-term, strain-resolved (e.g., shotgun metagenomic) analyses will be required to establish whether VSL#3® induces stable microbiota remodeling or transient enrichment of the supplemented organisms.

Persistent immune activation and chronic low-grade inflammation have been increasingly implicated in long COVID pathophysiology [6, 46, 47]. In our cohort, VSL#3® supplementation was associated with reduced circulating levels of selected pro-inflammatory and immune-related cytokines, including IL1α, IL6, IL7, IFNα, and G-CSF, together with remodelling of immune cell populations characterized by increased CD8^+^ T cells and CD68^+^ macrophages and reduced CD66b+ granulocytes (Figures 4C,D). Of note, these cytokine changes were observed as within-group variations over time and did not reach statistical significance when compared with the placebo arm (Figures 4A,B); they should therefore be interpreted with caution as exploratory, hypothesis-generating findings. Correlation analyses additionally identified associations between health-associated bacterial taxa and immune regulatory markers (Figures 4E,F), supporting the possibility that microbiota-targeted interventions may contribute to modulation of persistent immune dysregulation in long COVID.

The microbiota-gut-brain axis has also emerged as a potential contributor to fatigue and neurocognitive symptoms in long COVID and related post-infectious chronic conditions [18, 19, 48–51]. In our study, fecal and serum neuromodulator levels showed high inter-individual variability and no consistent treatment-associated changes over time (Figures 5A–C). However, exploratory correlation analyses identified coordinated associations among microbiota-derived metabolites, health-associated bacterial taxa, immune-related markers, and clinical outcomes in responder patients (Figures 5D,E), suggesting the existence of broader microbiota–immune–metabolic interaction patterns associated with symptom improvement.

This study has several strengths, including the randomized double-blind placebo-controlled design and the integrated clinical, microbiota, immune, and metabolomic analyses. However, the relatively small cohort size, the limited duration of the post-treatment follow-up, the exploratory nature of the multi-omic analyses, and the lack of vaccination-status stratification represent important limitations. In addition, we were unable to assess whether the response to VSL#3® differed according to the presence of gastrointestinal symptoms during or after COVID-19, as the number of patients reporting these symptoms was too small to allow meaningful subgroup analyses. Future studies with larger cohorts should specifically investigate whether gastrointestinal manifestations identify a subset of Long COVID patients who may derive greater benefit from microbiota-targeted interventions. In conclusion, VSL#3® supplementation improved fatigue and selected quality-of-life parameters in patients with long COVID, with benefits persisting after treatment discontinuation. These clinical improvements were associated with coordinated changes involving gut microbiota composition, immune-related pathways, and microbiota-associated metabolic signatures, supporting further investigation of microbiota-targeted interventions in long COVID.

## Summary table

### What is known about this topic

- Long COVID is frequently associated with persistent fatigue and impaired quality of life.
- Gut microbiota dysbiosis and immune dysregulation are implicated in long COVID and linked to symptom persistence.
- Microbiota-targeted approaches such as probiotics are proposed for long COVID, but controlled clinical evidence is scarce.

### What this work adds

- VSL#3® significantly improved fatigue compared with placebo in patients with long COVID.
- Clinical benefit persisted after treatment discontinuation and was accompanied by improvements in selected patient-reported outcomes.
- Integrated multi-omics linked microbiota-derived metabolites and immune markers to clinical response.

This work represents an advance in biomedical science because it provides randomized placebo-controlled evidence supporting microbiota-targeted interventions for long COVID and identifies biological pathways associated with clinical improvement.

## Data Availability

The datasets presented in this study can be found in online repositories. The names of the repository/repositories and accession number(s) can be found below: https://www.ebi.ac.uk/ena, PRJEB78610.
