# Supplementary material for: VSL#3® supplementation improves fatigue in long COVID: results from the DELong#3 randomized placebo-controlled trial
Source: Br J Biomed Sci. 2026 Jul 27;83:16993. doi: 10.3389/bjbs.2026.16993 (PMC13455643; doi:10.3389/bjbs.2026.16993)
Supplement: Supplementary file 1 [file Supplementaryfile1.docx]

**Supplementary Methods**

**Questionnaires**

**Chalder Fatigue Scale (CFS):** CFS consists of 11 items assessing physical and mental fatigue. Fatigue was considered clinically relevant for scores ≥4/11 using the dichotomous scoring system. In the Likert scoring system, a total score ≥11/33 indicated clinically relevant fatigue (1).

**Hospital Anxiety and Depression Scale (HADS):** HADS consists of 14 items, including 7 items evaluating anxiety (HADS-A) and 7 evaluating depression (HADS-D). Each item is scored from 0 to 3. Final scores for each domain range from 0 to 21, with values ≥11 considered suggestive of clinically relevant anxiety or depression (2).

**Short Form-36 Health Survey (SF-36):** SF-36 consists of 36 items evaluating eight domains: physical functioning, role limitations due to physical problems, bodily pain, general health perception, vitality, social functioning, role limitations due to emotional problems, and mental health. Raw scores were transformed into standardized scores ranging from 0 to 100 for each domain, with higher scores indicating better health-related quality of life (3).

**Symptom Checklist-12 (SCL-12):** SCL-12 includes 12 questions evaluating somatic symptoms, including headache, dizziness, nausea, chest pain, lower back pain, muscle soreness, dyspnea, hot or cold spells, paresthesia, lump in the throat sensation, weakness, and heaviness in the limbs. Each symptom was scored according to the degree of distress experienced during the previous week on a scale ranging from 0 (“not at all”) to 4 (“extremely”). Raw scores were converted into standardized area T-scores based on a non-psychiatric normative population (4).

**Karnofsky Performance Status (KPS):** KPS consists of an 11-point scale corresponding to percentage values ranging from 100% (normal condition without symptoms) to 0% (death). The scale evaluates physical abilities, self-care, mobility, and the need for assistance (5).

**Visual Analogue Scale (VAS):** VAS is a subjective rating scale used to assess patients’ overall perception of well-being. The scale consists of a horizontal line with descriptive anchors at each end representing poor and excellent health conditions. Patients were asked to indicate their perceived health status by placing a mark on the line. The distance from the left end of the scale to the patient’s mark was measured to obtain a quantitative score .

**Structured Assessment of Gastrointestinal Symptoms (SAGIS):** SAGIS questionnaire includes 22 gastrointestinal symptoms scored on a five-point Likert scale ranging from 0 (“no problem”) to 4 (“very severe problem”). Symptoms were grouped into five domains: abdominal pain/discomfort, diarrhea/incontinence, gastroesophageal reflux/regurgitation, nausea/vomiting, and constipation. Scores for each domain were calculated as the arithmetic mean of the symptoms included within the corresponding domain. The SAGIS questionnaire additionally included open-ended questions regarding the patients’ main health concerns and the presence or absence of selected non-gastrointestinal symptoms, including headache, back pain, sleep disturbances, chronic fatigue, anxiety, and depression (6).

**Clinical data analysis**

The distributions of questionnaire scores were represented using violin plots. Differences in questionnaire scores between baseline (T0) and week 4 (T4) in placebo- and VSL#3®-treated patients were analyzed using two-way ANOVA in GraphPad Prism (v10.2.3, GraphPad Software, Boston, MA, USA).

For CFS, HADS-A, HADS-D, VAS, SCL-12, and SAGIS-derived scores, values were transformed by subtracting the observed score from the maximum achievable score for the corresponding questionnaire, so that higher scores consistently reflected better clinical conditions across all questionnaires.

For each patient and questionnaire, score variations between T4 and T0 were calculated in R environment (v4.4.1). Percentage increase was calculated as the difference between the median T4–T0 variation observed in VSL#3®-treated patients and the median variation observed in placebo-treated patients, divided by the maximum obtainable score for the corresponding questionnaire.

Differences between groups were assessed using the Mann-Whitney U test, and effect sizes were additionally calculated. False discovery rate (FDR) correction was applied to multiple comparisons. FDR values obtained from the Mann-Whitney U test were plotted against percentage score increases after VSL#3® supplementation compared with placebo. Questionnaires with FDR <0.05 were highlighted in the resulting plots.

Bar plots reporting effect sizes and corresponding FDR-adjusted p-values for T4-T0 score variations between VSL#3® and placebo groups were generated using GraphPad Prism.

**References**

1.	Chalder T, Berelowitz G, Pawlikowska T, Watts L, Wessely S, Wright D, et al. Development of a fatigue scale. J Psychosom Res. 1993;37(2):147–53. doi:10.1016/0022-3999(93)90081-p PubMed PMID: 8463991.

2.	Zigmond AS, Snaith RP. The hospital anxiety and depression scale. Acta Psychiatr Scand. 1983 Jun;67(6):361–70. doi:10.1111/j.1600-0447.1983.tb09716.x PubMed PMID: 6880820.

3.	Apolone G, Mosconi P. The Italian SF-36 Health Survey: translation, validation and norming. J Clin Epidemiol. 1998 Nov;51(11):1025–36. doi:10.1016/s0895-4356(98)00094-8 PubMed PMID: 9817120.

4.	Derogatis LR, Cleary PA. Confirmation of the dimensional structure of the scl-90: A study in construct validation. Journal of Clinical Psychology. 1977;33(4):981–9. doi:10.1002/1097-4679(197710)33:4<981::AID-JCLP2270330412>3.0.CO;2-0

5.	Péus D, Newcomb N, Hofer S. Appraisal of the Karnofsky Performance Status and proposal of a simple algorithmic system for its evaluation. BMC Med Inform Decis Mak. 2013 Jul 19;13:72. doi:10.1186/1472-6947-13-72 PubMed PMID: 23870327; PubMed Central PMCID: PMC3722041.

6.	Koloski NA, Jones M, Hammer J, von Wulffen M, Shah A, Hoelz H, et al. The Validity of a New Structured Assessment of Gastrointestinal Symptoms Scale (SAGIS) for Evaluating Symptoms in the Clinical Setting. Dig Dis Sci. 2017 Aug;62(8):1913–22. doi:10.1007/s10620-017-4599-6 PubMed PMID: 28551709.

**Supplementary Tables**

**Supplementary Table 1**: Analytes in the 45-plex Luminex assay

|  | **Analyte** |  | **Analyte** |  | **Analyte** |
| --- | --- | --- | --- | --- | --- |
| **Cytokines** | IL-2 | **Chemoattractors** | CXCL1 (GROa) | **Signalling** | CD40L |
|  | IL-7 |  | CXCL2 (GROb) |  | PDL1 |
|  | IL-15 |  | CXCL8 (IL-8) |  |  |
|  | IL-10 |  | CXCL10 (IP-10) |  |  |
|  | IL-33 |  | CX3CL1 (Fraktalkine) |  |  |
|  | IL-4 |  | CCL2 (MCP-1) |  |  |
|  | IL-5 |  | CCL3 (MIP1a) |  |  |
|  | IL-13 |  | CCL4 (MIP-1b) |  |  |
|  | IL-1a |  | CCL5 (Rantes) |  |  |
|  | IL-1b |  | CCL11 (Eotaxin) |  |  |
|  | IL-1Ra |  | CCL19 (MIP3b) |  |  |
|  | IL-12p70 |  | CCL20 (MIP3a) |  |  |
|  | IFNa |  |  |  |  |
|  | IFNb | **Growth factors** | FLT3L |  |  |
|  | IFNg |  | IL-3 |  |  |
|  | TNFa |  | G-CSF |  |  |
|  | TRAIL |  | GM-CSF |  |  |
|  | GRZb |  | TGFa |  |  |
|  | IL-6 |  | EGF |  |  |
|  | IL-17A |  | PDGFaa |  |  |
|  | IL-17F |  | PDGFab/bb |  |  |
|  | IL-22 |  | VEGF |  |  |
|  | IL-23 |  | FGF basic |  |  |

**Supplementary Table 2**: Antibodies used for multiparametric FACS analysis

|  | Clone |
| --- | --- |
| CD3 | UCHT1 |
| CD4 | A161A1 |
| CD8 | SK1 |
| IL10 | JES3-19F1 |
| IFNg | 4S.B3 |
| Il17 | eBio64DEC17 |
| IL22 | 2G12A41 |
| CD11c | 3.9 |
| CD66b | G10F5 |
| CD68 | eBIoY1/82A |

**Supplementary Table 3**: Attribution of ASVs

| **ASV** | **BLAST result** |  |
| --- | --- | --- |
| ASV_434 | uncultured bacterium |  |
| *Bifidobacterium_ASV_40* | *Bifidobacterium animalis subsp. lactis* |  |
| *Lactobacillus_ASV_634* | *Lactobacillus plantarum/Lactobacillus sp./Lactiplantibacillus argentoratensis/Lactiplantibacillus pentosus* |  |
| *Bifidobacterium_ASV_2663* | *Bifidobacterium sp./Bifidobacterium animalis subsp. Lactis* |  |
| *Lactobacillus_ASV_257* | *Lactobacillus acidophilus* |  |
| Bacteroides_ASV_2422 | uncultured bacterium/uncultured Clostridium sp./uncultured Bacteroidales bacterium/uncultured Morganella sp. |  |
| Ruminococcus_ASV_78 | Ruminococcoides bili |  |
| Barnesiella_ASV_104 | uncultured bacterium/uncultured Barnesiella sp. |  |
| ASV_185 | uncultured bacterium/uncultured Eubacterium sp./uncultured Lachnospiraceae bacterium |  |
| Coprococcus_ASV_301 | Coprococcus sp. |  |
| Clostridium XlVa_ASV_182 | [Clostridium] hylemonae |  |
| *Lactobacillus_ASV_334* | *Lactiplantibacillus plantarum/Lactiplantibacillus sp./Lactiplantibacillus argentoratensis/Lactiplantibacillus fabifermentans/Lactiplantibacillus pentosus* |  |
| Bacteroides_ASV_114 | Phocaeicola massiliensis/Bacteroides sp. |  |
| *Streptococcus_ASV_21* | *Streptococcus thermophilus* |  |
| ASV_281 | uncultured prokaryote/uncultured anaerobic bacterium/uncultured Kineothrix sp. |  |
| Leuconostoc_ASV_1870 | Leuconostoc carnosum |  |
| Bacteroides_ASV_213 | Bacteroides koreensis/Bacteroides ovatus/Bacteroides sp. |  |
| Catenibacterium_ASV_272 | Catenibacterium mitsuokai |  |
| Alloprevotella_ASV_186 | uncultured Bacteroides sp./uncultured Alloprevotella sp. |  |
| Prevotella_ASV_81 | uncultured bacterium/uncultured Bacteroidetes bacterium/Segatella copri/uncultured Segatella sp. |  |
| Barnesiella_ASV_3763 | [uncultured bacterium/uncultured Barnesiella sp.](https://www.ncbi.nlm.nih.gov/Taxonomy/Browser/wwwtax.cgi?id=77133) |  |
| Bacteroides_ASV_8 | Bacteroides uniformis strain/Uncultured Bacteroides sp. |  |
| Lachnospiracea_incertae_sedis_ASV_2353 | Anaerobutyricum soehngenii/Anaerobutyricum hallii strain/unclutured Eubacterium sp. |  |
| Bacteroides_ASV_134 | uncultured bacterium/Phocaeicola sp./uncultured Bacteroides sp. |  |
| Bacteroides_ASV_75 | Bacteroides uniformis/uncultured Bacteroides sp. |  |
| Clostridium sensu stricto_ASV_46 | Clostridium celatum/Clostridium disporicum/ Clostridium saudiense |  |
| ASV_121 | uncultured bacterium/uncultured Acetivibrio sp./uncultured Eubacteriales bacterium/ |  |
| Odoribacter_ASV_90 | uncultured Odoribacter sp./Odoribacter splanchnicus |  |
| ASV_801 | uncultured bacterium/uncultured Christensenellaceae bacterium |  |
| Anaerostipes_ASV_219 | uncultured bacterium/Anaerostipes hadrus/uncultured Anaerostipes sp. |  |
|  | |  |

**Supplementary Figures**

***Figure S1. Health-related quality of life assessed by SF-36.*** *Change in SF-36 domain scores from baseline (t0) to the end of treatment (t4) in placebo (blue) and VSL#3®-treated (red) patients. Statistical analyses were performed by two-way ANOVA. *p < 0.05; **p < 0.01; ****p < 0.0001*

***Figure S2. Gastrointestinal symptoms assessed by SAGIS.*** *Change in SAGIS domain scores from baseline (t0) to the end of treatment (t4) in placebo (blue) and VSL#3®-treated (red) patients. Statistical analyses were performed by two-way ANOVA. *p < 0.05; **p < 0.01; ****p < 0.0001*

***Figure S3. Mental health and functional outcomes.*** *Change in HADS (A: anxiety; D: depression), SCL-12, KPS, and VAS scores from baseline (t0) to the end of treatment (t4) in placebo (blue) and VSL#3®-treated (red) patients. Statistical analyses were performed by two-way ANOVA. *p < 0.05; **p < 0.01; ****p < 0.0001.*

***Figure S4. Predicted functional changes in the gut microbiota following VSL#3® supplementation.*** *(A) Reconstructed metabolic map highlighting pathways enriched in VSL#3®-treated patients (red). Statistical analysis has been performed by Mann Whitney test, two tailed. (B) Effect size and FDR of the KEGG ortholog pathway (ko00260: glycine, serine and threonine metabolism) enriched in VSL#3®-treated patients.*
